# CherryChèvre: A fine-grained dataset for goat detection in natural environments

**DOI:** 10.1038/s41597-023-02555-8

**Published:** 2023-10-11

**Authors:** Jehan-Antoine Vayssade, Rémy Arquet, Willy Troupe, Mathieu Bonneau

**Affiliations:** 1 INRAe - ASSET, Animal Genetic, 97170 Petit-Bourg, Guadeloupe; 2 INRAe - UE PTEA, 97170 Petit-Bourg, Guadeloupe

**Keywords:** Scientific data, Agriculture, Computer science

## Abstract

We introduce a new dataset for goat detection that contains 6160 annotated images captured under varying environmental conditions. The dataset is intended for developing machine learning algorithms for goat detection, with applications in precision agriculture, animal welfare, behaviour analysis, and animal husbandry. The annotations were performed by expert in computer vision, ensuring high accuracy and consistency. The dataset is publicly available and can be used as a benchmark for evaluating existing algorithms. This dataset advances research in computer vision for agriculture.

## Background & Summary

Agriculture is a crucial industry that holds immense importance in ensuring global food security and sustaining the economy. Among the most commonly kept livestock animals worldwide, goats are known for their adaptability to various environmental conditions^1^, high productivity and quality for milk^2^ and meat^3^. However, researchers are faced with the challenge of effectively detecting and tracking goat herds to analyze their behavior. It could be achieved trough visual observation, a time-consuming and error-prone method for which it is difficult to obtain fine-grained data on the precise movements and behaviors of goats. One alternative is to use a GPS unit^4^, which requires handling the animal and may be inaccurate in certain isolated location, especially if one intends to monitor at pasture scale.

Automated methods for detecting and tracking animals through computer vision techniques can mitigate some of the challenges of behavior analysis. However, the development of these techniques heavily relies on the availability of high-quality datasets for training and evaluation. Despite the existence of livestock detection datasets, there is still a dearth of comprehensive, top-notch datasets especially for goat detection. Moreover, the accuracy of detection is closely tied to the quality of the dataset^5^. Hence, the creation of a high-quality dataset for goat detection is crucial for improving the performance of computer vision algorithms in the field of precision agriculture.

To address this gap, we introduce the first dataset for goat detection that contains 6160 annotated images of goats captured under varying environmental conditions. The images were collected from field surveys. Each image was annotated with bounding boxes around the goat’s body. All bounding boxes stick at pixel level around body (when possible: feet on leaves, fur that create blur, etc). The annotations were performed by an expert in the field of computer vision, ensuring high accuracy and consistency. The dataset contains images of goats in various poses and orientations, including standing, grazing, and lying down. The images were captured under different lighting conditions, including few bright sunlight and low light. The dataset is intended for use in developing machine learning algorithms for goat detection, with potential applications in precision agriculture, wildlife conservation, animal welfare, and animal husbandry. The dataset can also be used as a benchmark for evaluating existing detection methods.

## Existing datasets for object detection

### Common objects in context

**Fig. 1:**
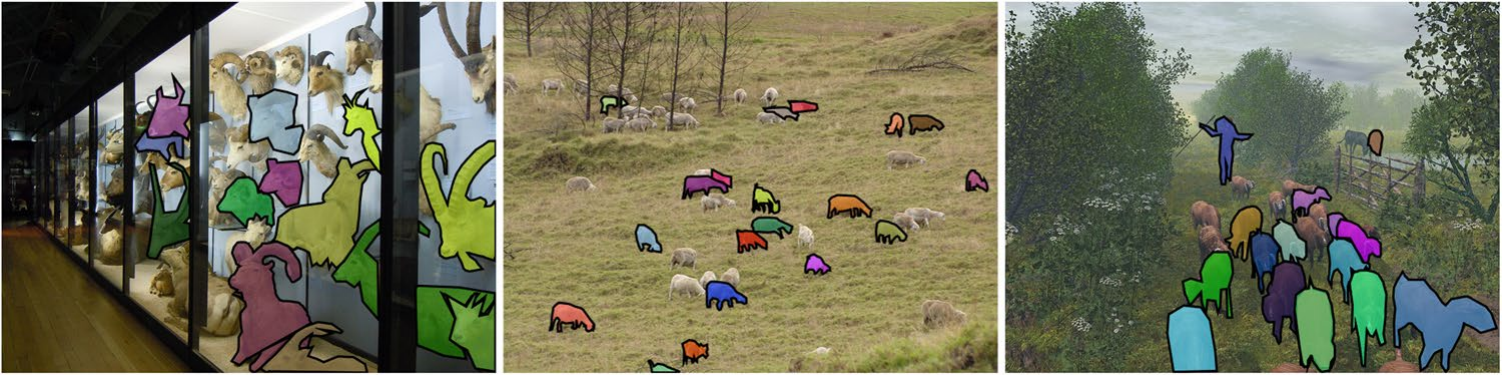
Example of images featuring sheep from the COCO dataset.

Microsoft COCO^6^ is a large-scale image recognition, segmentation, and captioning dataset that contains more than 328,000 images with over 2.5 million object instances labeled and segmented across 80 different categories. COCO is one of the most widely used benchmark dataset for object detection and instance segmentation, and it has been used for animal detection as well. Researchers have used COCO to train and evaluate animal detection models, including detecting animals in the wild, on farms, and in zoos. The animal categories in COCO include bird, cat, dog, horse, sheep, cow, and elephant, among others, but no goat class. The coco dataset include bounding boxes, segmentation and other information as depicted in Fig. 1.

### PASCAL (Visual Object Classes)

**Fig. 2:**
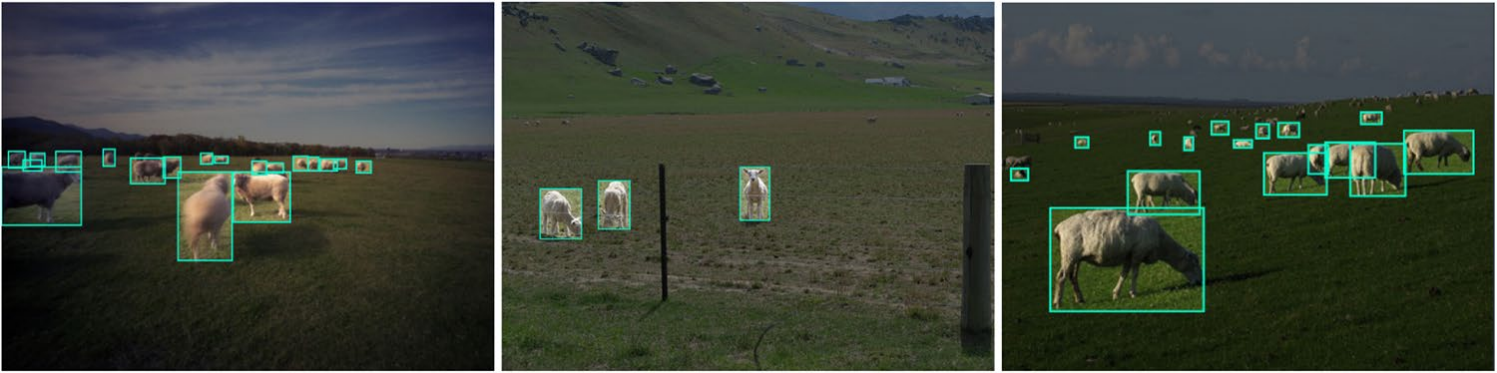
Example of images featuring sheep from the PASCAL VOC dataset.

PASCAL VOC^7^ is a benchmark dataset and competition for object detection and recognition in natural images. It includes a set of image classification, detection, and segmentation challenges, with the goal of advancing the state-of-the-art in computer vision research. The dataset includes annotated images of 21 objects classes, including animals, such as cat, cow, dog, horse, and sheep, but no goat class. Figure 2 shows some examples from the dataset.

### Roboflow

**Fig. 3:**
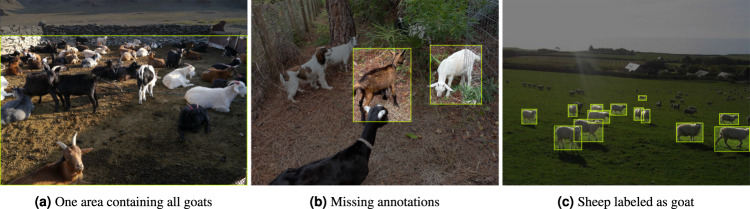
Incorrect annotation among different goat datasets in Roboflow.

Roboflow is a computer vision startup that provides tools and services for training, deploying, and improving computer vision models^8^. One of their initiatives is the Universe project, which is a collection of datasets curated from “open-source” image and video datasets. The project aims to provide a single place where developers and researchers can access a diverse set of computer vision datasets. Within Roboflow, only five datasets have a goat class. Figure 3 shows some pictures taken from different Roboflow goat datasets that exhibit erroneous annotation.

### Limitation of existing dataset

The COCO dataset has been criticized^5^ for its inaccurate annotations and low quality. One of the reasons for the inaccuracy is that the annotations are done by crowd-workers who have varying levels of expertise in object detection. Additionally, some objects may be missed or incorrectly labeled due to the complexity of the scene or the object’s appearance. This is illustrated in Fig. 1, where several number of false negatives are present. Similar to the COCO dataset, PASCAL VOC also suffers from limitations in terms of accuracy^9^. While the bounding box alignment may seem better than COCO, there still exist a wide number of false negatives, as shown in Fig. 2. Finally as illustrated in Fig. 3, Roboflow datasets are largely affected by quality issues, such as inaccuracy of bounding box fit, resulting in objects being cropped, missed entirely or fused on the whole picture. Finally, some datasets may have licensing restrictions that could limit their use in certain case.

To summarize, it is critical to recognize potential quality issues in available datasets, such as inaccurate bounding box fitting and licensing issues. Most of the datasets available online for livestock monitoring are not reliable sources of data, particularly for goat detection. Despite the lack of datasets dedicated to goat detection, pre-training object detection models on these datasets can still be beneficial to provide a good initialization.

### Importance of dataset quality

The quality of a dataset plays a crucial role in predicting the accuracy of a machine learning algorithm trained on it^10,11^. In the case of goat detection, a high quality dataset such as the one in this work, with accurate and consistent bounding box annotations, diverse and representative images, and minimal noise and errors, can greatly improve the accuracy and robustness of the trained object detector^5^. This can be considered as data-centric approach.

On the other hand, a low quality dataset with incorrect or inconsistent annotations, limited or biased images, and noisy or erroneous data can negatively affect the accuracy and generalization ability of the trained detector^12^. This is because the detector learns from the patterns and features present in the training data, and if the data is of poor quality, the detector may learn incorrect or irrelevant patterns, resulting in poor performance on new, unseen data^13^. Even a small proportion of False Positive or False Negative could greatly affect models accuracy. This can be strengthened by the selected loss function, like Focal loss^14^ used from YOLO v3^15^ to YOLO v8^16^.

In addition, inconsistencies in the annotation of goat bodies can lead to the detection of inaccurate bounding boxes in videos, making animal tracking difficult. In such cases, the bounding boxes may shift or shake relative to reality, leading to inaccuracies in tracking. This problem can also complicate accurate analysis of goat behavior, as a goat that is not moving can also be detected unstably. In addition, animals that are close to each other may be misdetected or merged due to the fact that the detector has been trained on a data set with these examples.

Therefore, it is important to ensure that the training dataset is of high quality and representative of the real-world scenarios where individuals are present. This can help improve the accuracy and robustness of the detector and enable more accurate analysis and monitoring of goat behavior in different settings. It also means that a high quality dataset allows greater generalizability than a dataset with more data but lower quality, so quality should always be prioritized over quantity when possible^11^.

## Methods

In this section, we present all the data acquisition sources each representing a subset of the dataset. Each subset is presented from experimental plots to sensor characteristics. Finally, acquired images were annotated, using VGG Image Annotator (VIA)0^17^. Note that animals were raised under normal conditions, most of them under a tropical climate.

### Cross-call

The Crosscall Trecker X2 is a rugged smartphone that was utilized to capture a total of 297 images at various dates and times, allowing for a diverse range of lighting and environmental conditions. This device is equipped with advanced sensors, allowing to capture high-quality images. The rear camera of the Trecker X2 is a 12-megapixel sensor that features an aperture of *f*/2.0, capturing clear and sharp images even in low light conditions. Similar to any modern smartphones, it includes an auto-focus system that guarantees images are always in focus. Table 1 displays the quantity of annotated images captured at the INRAE-Duclos facility in Guadeloupe, French West Indies, for all dates except the initial one. The first date was taken near Albiez-Montrond, 73300, Albiez-Montrond, and a few images were also taken near Tesq, 12210 Montpeyroux, both in France in 2020. The dataset contains a mix of white sheep, goats, and mainly Creole sheep, that are mostly practically indistinguishable in appearance to European goats.

**Table 1:** Number of annotated images by date for the Cross-call device.

Date	Images
17/04/2020	20
19/12/2022	49
01/13/2023	15
31/01/2023	20
03/02/2023	17
06/02/2023	18
07/02/2023	44
13/02/2023	22
14/02/2023	55
15/02/2023	30

### Phantom3

In a previous study^18^, Creole goats grazing on two distinct pastures (G1 + G2) were recorded using a Phantom3 UAV drone equipped with a 12-megapixel camera sensor. The camera had a 94-degree field of view lens, which enabled wide-angle shots, and images were captured at a maximum resolution of 4000 × 3000 *px*. The study was conducted over four successive days in April 2017 at the INRA-PTEA farm (16° 2  N; 61° 2  W), and a total of 47 images were re-annotated to include kids. To account for the small size of the animals and the large image size, each image was subdivided into smaller ones, resulting in 696 images with dimensions of 1000 × 750 *px*. Additionally, two videos were recorded on April 13, 2017, using the same drone and providing different perspectives of the pasture. Table 2 show the number of acquisition per pasture (G1 + G2) and date.

**Table 2:** Number of annotated images by date for the Phantom3.

Source	Date	Images
G1	10/04/2017	299
G2	10/04/2017	281
G1	11/04/2017	35
G2	11/04/2017	46
G1	12/04/2017	35
Videos	13/04/2017	150

### Time-lapse camera

We used construction time-lapse cameras (TLC2000 pro, year 2018, brand Brinno) on various previous studies^19–22^. These cameras record at 1.3 Mpx with a resolution of 1280 × 720 *px* using jpeg compression. Those studies are presented bellow:

The initial research conducted as part of a master’s thesis^19^ set up different experimental plots to study the detection and tracking of goats and sheep. The first subset includes seven Creole sheep raised indoor with identical reddish coats. The second data set contains one Creole sheep with a brown coat. The third contains nine goats near the camera, six of them have dark coats, while the others have red coats. Additionally, few goats appear far away and have been annotated.

As a result of this initial research, the proposed framework was improved and rigorously evaluated in the article^20^. The authors of the article built upon previous research by collecting additional data in natural environments with different lighting conditions. Once again, the previously defined framework was refined and tested by monitoring two goat herds under farm-like conditions. One time-lapse camera monitored an area of approximately 20 × 20 *meter*, and multiple cameras were combined to monitor the entire pasture^21^.

As part of the experimental design to evaluate fecal avoidance in goats^22^, four male Creole goats were selected to ensure color diversity. Color selection was intended to facilitate the identification and tracking of goats during the experiment by allowing them to be followed through image classification.

All of these previous research have resulted in a large amount of images being collected. Some of the data was extracted and carefully re-annotated. Table 3 summarizes the number of images annotated within this subset:

**Table 3:** Number of annotated images by date for the TLC2000 device.

Date	Study	Images
2018	^19^	140
2020	^20,21^	1446
2022	^22^	784

### Tracking series

A CCTV camera (ENEO - IPD-75M2713M5A) with a resolution of 2592 × 1944 *px* was used to capture 17 videos of goats grazing on pasture in 2022, over 5 different days, to develop a tracking algorithm^23^. Each movie has been subsampled to obtain 98 images, with 50 showing non-overlapping goats and 50 featuring at least 2 goats overlapping. These videos were recorded at two different places of the INRAE-PTEA facility in Guadeloupe, French West Indies. In early 2023, 6 new videos were generated over 3 separate days at the experimental plot in Duclos, featuring male goats with distinct coat colors: dark, white, russet, and dark-russet. Each goat was outfitted with a collar, with red, yellow, orange, and blue colors, respectively, to attach an accelerometer. Eight additional videos were also captured at Gardel in early 2023 during 4 different days. As before, the same sub-sampling method was used. The number of annotated images for each date are presented in Table 4.

**Table 4:** Dates, location and number of annotated images, using the ENEO camera.

Date	Location	Images
12/04/2022	Duclos	98
22/04/2022	Duclos	687
26/04/2022	Duclos	197
17/02/2023	Duclos	136
23/02/2023	Duclos	280
24/02/2023	Duclos	97
02/05/2022	Gardel	196
16/06/2022	Gardel	489
14/03/2023	Gardel	146
16/03/2023	Gardel	33
17/03/2023	Gardel	25
24/03/2023	Gardel	25

### External

It’s a subset that combines images from two sites. One site, named Mosar, is located at the INRAE UMR 791 Modélisation Systémique Appliquée aux Ruminants experimental installation (Grignon, France). This subset includes indoor European goats and displays two indoor pens with eight animals each. The camera and feeding tray arrangement mainly captures goats from behind, and four 30-minute videos were sub-sampled. The second collaborator, named Ferlus, is located at the Experimental Unit FERLUS (10.15454/1.5572219564109097E12 - Lusignan, France) and consists of a small number of ground-level images of European goats in an external pasture. The image sizes are 1280 × 720 *px* and 4032 × 3024 *px* for the first and second subsets, respectively. Table 5 shows the number of annotated images for each acquisition.

**Table 5:** Date, location and number of annotated images for external sources.

Date	Location	Images
23/03/2022	Mosar-p1	151
23/03/2022	Mosar-p2	45
23/03/2022	Mosar-p3	45
23/03/2022	Mosar-p4	44
10/03/2016	Ferlus	14

## Data Records

The authors of this study have publicly released CherryChèvre dataset, which is available in VGG format at 10.57745/QEZBNA^24^ (subsets are available separately in dedicated TGZ archives for each of them) and in YOLO format with a train/validation/test split at 10.57745/4C03OG^25^. Since the YOLO format uses normalized coordinates, it may be beneficial to resize all images to the training size (e.g., 640 × 640 or 1280 × 1280) to optimize learning time. The dataset is licensed under https://spdx.org/licenses/etalab-2.0.htmlEtalab Open License 2.0. This License is designed to be compatible with any free license that requires at least a statement of authorship, including Open Government Licence, Creative Commons Attribution 2.0 CC-BY and Open Data Commons Attribution. This compatibility allows for extensive reuse and modification of the data, provided that the original authors are properly attributed.

## Technical Validation

This section provides a detailed overview of the proposed dataset, including statistics, showcases, and a performance evaluation. The dataset needs to be analyzed to understand its limitations and potential applications, and the performance evaluation results provide insight on the effectiveness of object detection applied to our dataset.

### Statistics of the dataset

CherryChèvre is composed of five distinct subsets and contains a total of 6160 images. These images were carefully annotated with 35381 unique bounding boxes. The number of bounding boxes varies across the subsets, as detailed in Table 6. The Timelapse and Tracking subsets contain 77 of the labeled images, showing that CherryGoat is well balanced for these two subsets but the others are less represented.

**Table 6:** Total count of annotated images and bounding boxes for each of the five subsets. The final column displays the ratio of bboxes to images, indicating the average density of goats per image.

Subset	Number of images	Number of bboxes	bboxe/img
Crosscall	290	2069	7.13
Phantom3	846	7520	8.89
External	297	3522	11.85
Timelapse	2327	11685	5.02
Tracking	2400	10585	4.41
Overall dataset	6160	35381	5.74

**Fig. 4:**
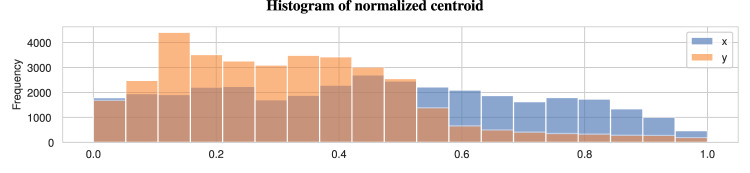
Normalized centroid distribution across the entire dataset. The presence of the sky and ground areas results in a less uniform sampling of goat positions along the vertical dimension (y).

Figure 4 displays the normalized centroid distribution for the entire dataset, obtained by dividing the bounding box position (centroid) by the size of the image. The first blue bin on the x-axis, ranging from 0.0 to 0.052, shows that 1770 bounding boxes appear near the left border of the image (between 0% and 5.2% of the image size). Similarly, the last blue bin shows 453 bounding boxes appear near the right side of the images. Thus Figure 4 highlights that the presence of sky and ground areas leads to uneven sampling of goat positions along the vertical dimension. To address this issue, data augmentation techniques^26^ can be used to add random displacement (20 40%) along this axis. This approach will facilitate sampling of all screen positions during the training phase.

**Fig. 5:**
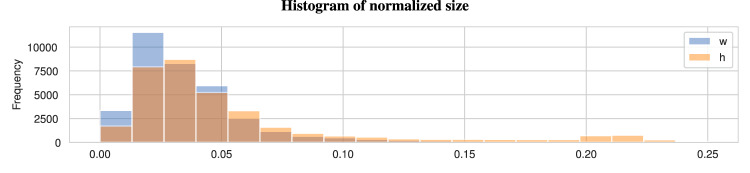
Distribution of normalized size, which includes both width and height, for the entire dataset. The results indicate that both dimensions are evenly distributed.

Figure 5 displays the distribution of the normalized size, which includes both width and height, for the entire dataset, obtained by dividing the bounding box size by the size of the image. The results indicate that both dimensions are evenly distributed. Moreover, it reveals that larger objects are less represented in the dataset. Therefore, models trained on this dataset may not detect a goat that appears in an entire image. However, medium and small-sized goats are well-represented, making the dataset suitable for monitoring goats in a wide area. For those who wish to detect goats in full screen, the present dataset can be reused by dividing the images into several pieces, each piece containing one or more individuals but excluding most of the backgrounds. This can also be implemented as a data augmentation technique^26^.

**Fig. 6:**
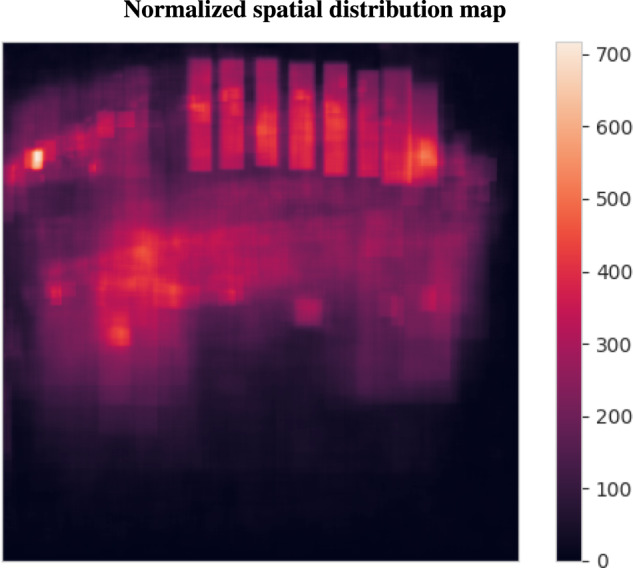
Spatial distribution of bounding boxes in the images across the dataset. The color indicates the density of bounding boxes, with white indicating a high density, red a medium density and black indicating a density near zero. The figure provides insight into the spatial distribution of the animals within the images and highlights the areas where the animals are most frequently present.

Figure 6, is presented to reinforce the previously mentioned observations. It showcases the total sum of normalized bounding boxes across the entire dataset, revealing that most of the annotations are concentrated in certain areas while there is a comparatively lower density of annotations towards the edges. This could be attributed to the placement of the fence and the fixed position and orientation of the camera in most cases. To address this issue, a simple vertical and horizontal flip could be implemented as a data augmentation technique, along with a random translation, to efficiently sample all screen positions.

The figures presented in this section provide important information about the dataset, offering significant insights into its properties and features. This information can be leveraged to optimize the training and testing phases for goat detection models, with data augmentation technics.

### Examples of annotated images

**Fig. 7:**
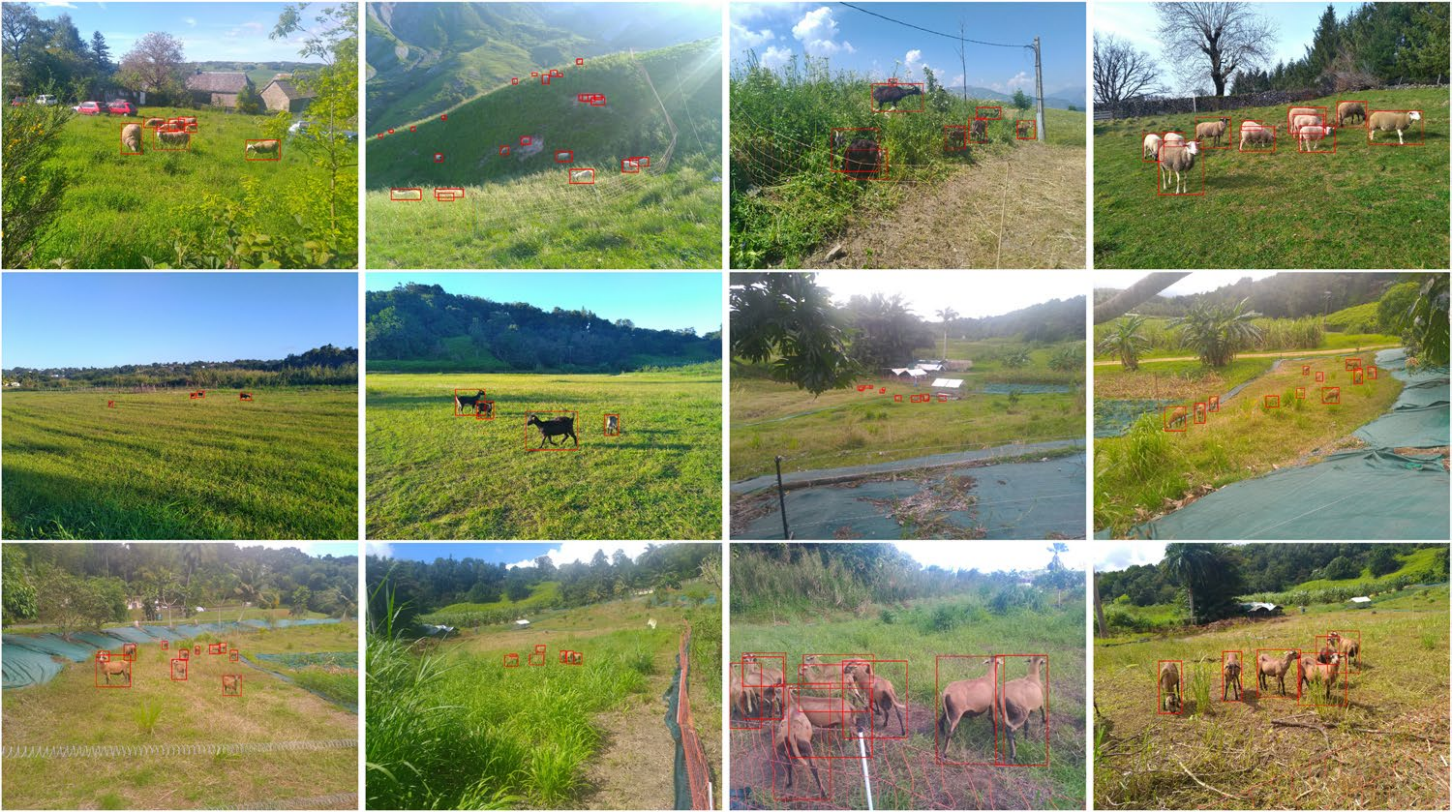
Example of annotated images for the Crosscall subset. The first line shows images near Albiez-Montrond and near Laguiole while the others was taken in INRAe Duclos. These images are very diverse, showing Creole sheep in high level of weed. These sheep are hard to distinguish from European goat.

**Fig. 8:**
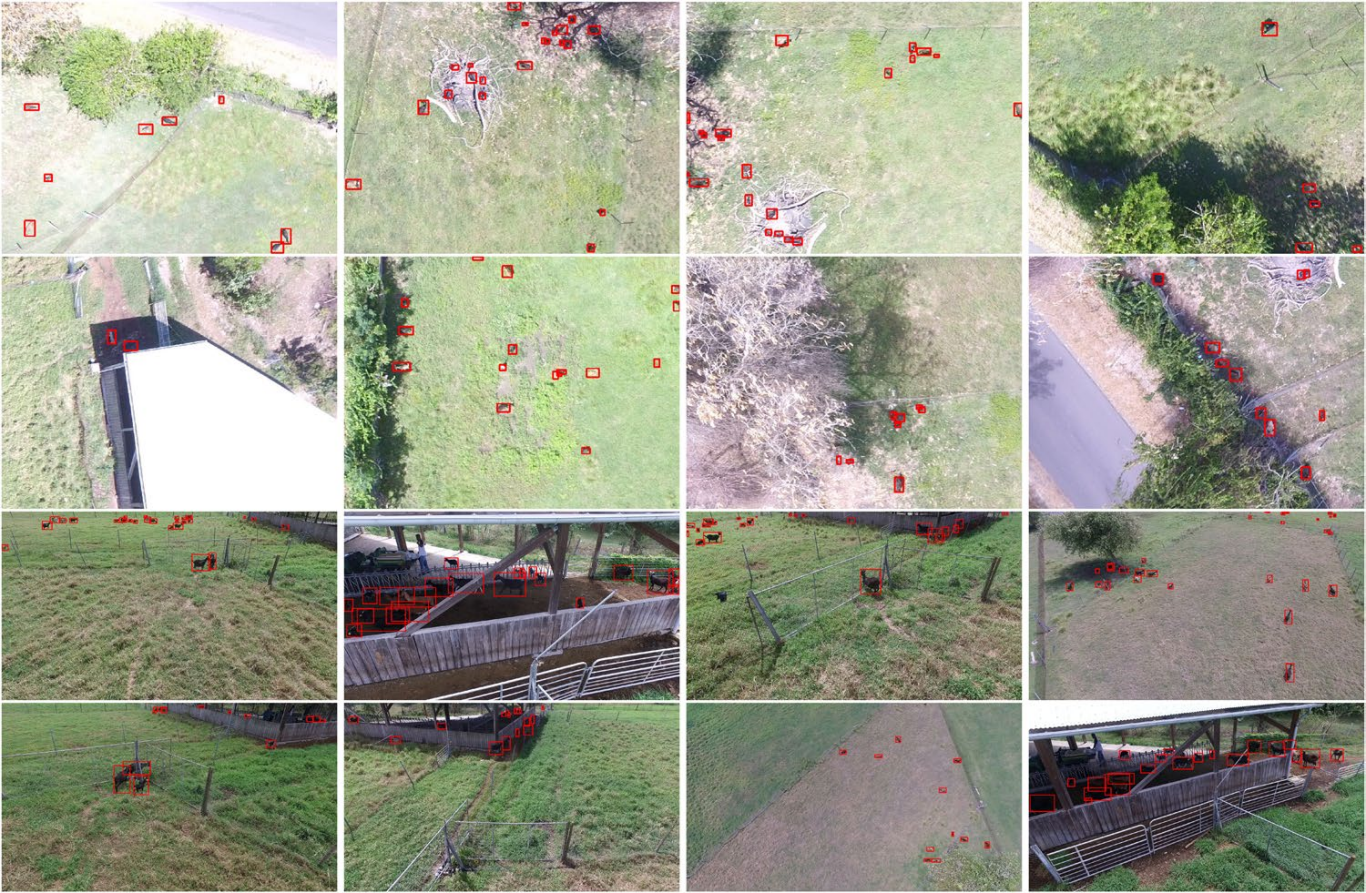
Example of annotated images for the Phantom3 subset. The first two lines feature goat captured from 22 meters above the ground, while the following two lines offer a variety of viewpoints from distant to close-up goats, as well as goats in interior environments, all captured by the flying drone.

**Fig. 9:**
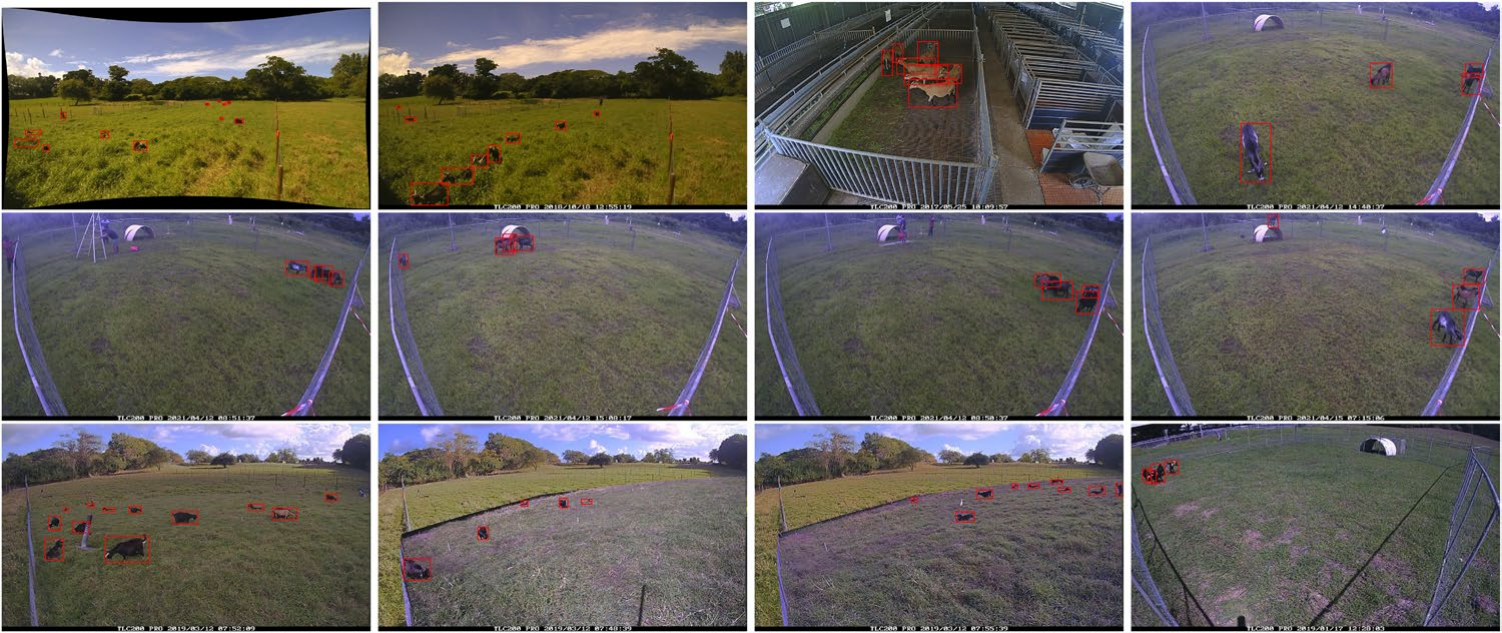
Example of annotated images for the Timelapse subset. These images are of lower quality, smaller and noisier. However, they have a large amount of individuals, and a lot of overlap between individuals. The angles of view, height of weeds, etc, are also different. This data set is therefore important, especially for those who wish to work with low resolution cameras, for wildlife conservation or theft prevention.

**Fig. 10:**
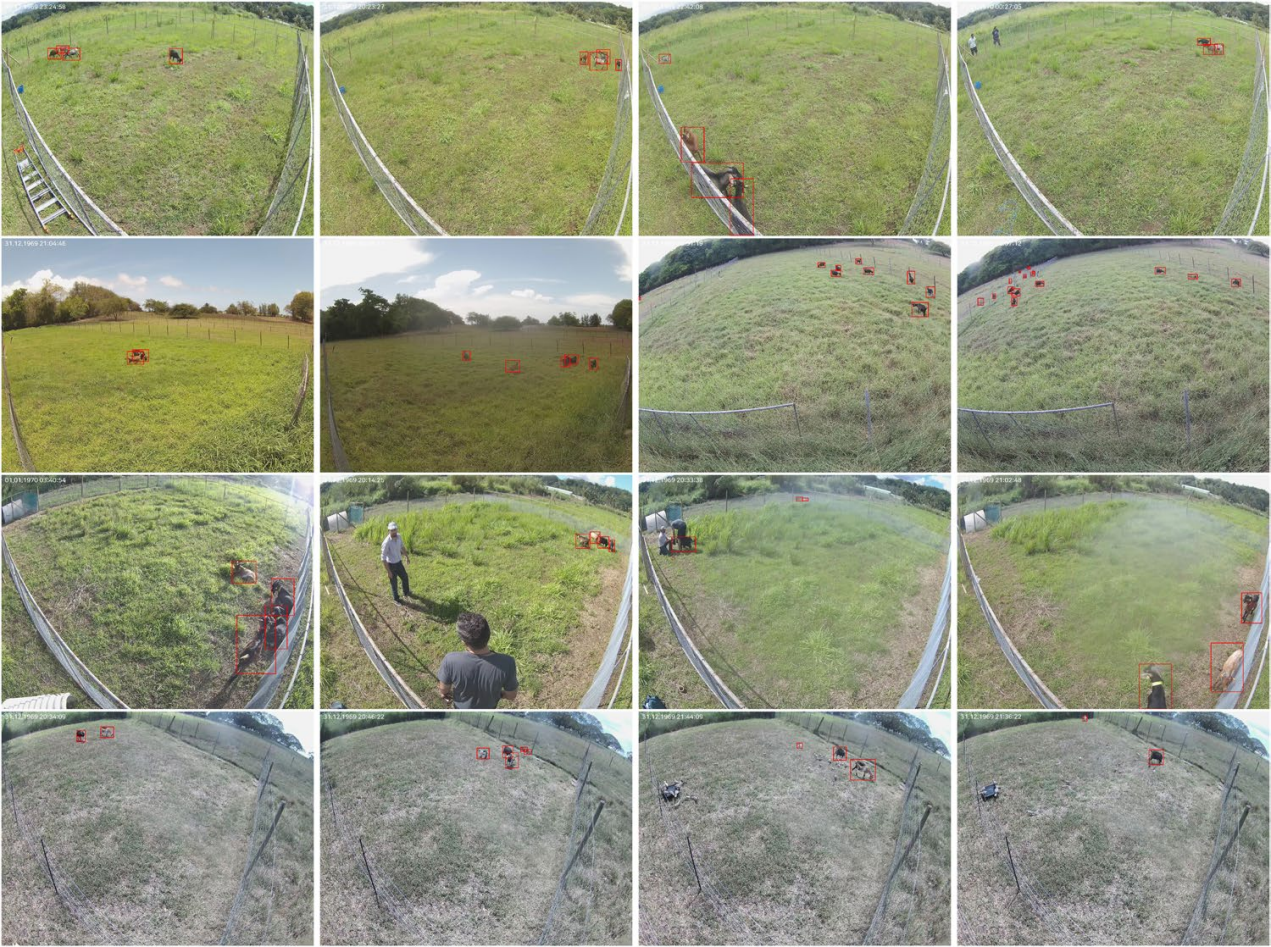
Example of annotated images for the Tracking subset. It contains high-quality annotated images, which ensures the best detection quality, which is critical for studying animal movement patterns, habitat usage, and behavior.

**Fig. 11:**
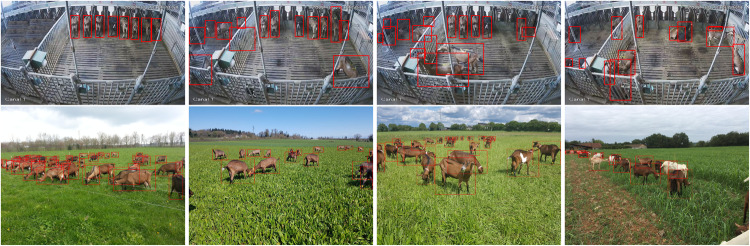
Example of annotated images for the External subset. It mostly showcases goats raised indoors, as seen in the first line which features goats proposed trough Mosar. The second line highlights outdoor grazing goats proposed trough Ferlus.

This section provides visual examples of the annotations made on images within the dataset. It showcases how bounding boxes were drawn around the goats and provides insight into the level of detail and accuracy of the annotations. By showing actual examples from the dataset, it allows the reader to better understand the quality and usefulness of the data for machine learning and computer vision tasks related to goat detection and tracking. This can be see in Figs. 7–11.

### Performance evaluation

In order to evaluate the performance of the proposed dataset for goat detection a YOLOv8 object detection model^16^ (yolov8x6 and yolov8l, respectively with 97.321.636 and 43.630.611 parameters) has been trained. To ensure a representative distribution of data, the entire dataset was randomly divided into training (80%, or 4907 images), validation (10%, or 616 images), and test (10%, or 637 images) sets. This split was carefully design to include each source of input (by date of acquisition) in the same proportion for training, validation and test. The models were trained using default hyper-parameters on an RTX 3060 12GB GPU. To accommodate for memory limitations, the batch size was adjusted to 2 and 12 for yolov8x6 and yolov8l, respectively. Also the number of epochs differ due to excessive learning time, 200 and 330 was respectively set for YOLOv8x6 and YOLOv8l. Finally data augmentation technics are used trough de YOLO training framework, such as random cropping, flipping, color distortion, noise, rotation and scaling.

The results of this experiment provide insight into the effectiveness of the proposed dataset and its potential when coupled with YOLOv8. The evaluation was done with an open-source tool^27^ that extract most relevant metrics. Such as “COCO Average Precision” and “COCO Average Recall” which measures the quality of object detection by comparing the predicted bounding boxes to the ground truth bounding boxes of the objects in the images. The Average Precision (AP) metric evaluates a model’s ability to identify relevant objects by measuring the percentage of True Positive detection. On the other hand, the Average Recall (AR) metric evaluates a model’s ability to detect all relevant cases by measuring the percentage of true positive detections among all relevant ground truths.

The evaluation of detection quality is measured using different IoU thresholds (0.05, 0.50, 0.95), which assess different levels of accuracy. In addition, objects of varying sizes (small, medium, and large) are evaluated using separate metrics. Small objects are those with an area smaller than 32 × 32, large objects are those greater than 96 × 96, and medium objects are those in between. Concerning the AR metric, the recall is computed for 1, 10 and 100 maximum detections, for all IoU threshold and then averaged respectively for AR1, AR10 and AR100. These metrics were computed for both models, YOLOv8x6 (as shown in Table 7) and YOLOv8l (as shown in Table 8).

**Table 7:** Performance evaluation for YOLOv8x6. The input size is 1280 × 1280.

COCO Average Precision			
metric	train	validation	test
AP05	84.64	68.06	69.18
AP50	97.97	92.54	93.59
AP75	93.69	76.80	79.64
APsmall	77.43	52.49	53.67
APmedium	88.22	75.52	75.91
APlarge	89.18	76.71	77.53
**COCO Average Recall**			
AR1	16.25	14.51	14.82
AR10	76.56	62.98	64.59
AR100	87.31	71.74	72.68
ARsmall	81.13	58.48	59.80
ARmedium	90.49	78.87	79.24
ARlarge	91.00	79.88	80.47

**Table 8:** Performance evaluation for YOLOv8l. The input size is 640 × 640.

COCO Average Precision	train	validation	test
metric			
AP05	81.70	63.21	63.14
AP50	92.98	87.66	87.58
AP75	87.67	71.52	71.54
APsmall	66.15	43.61	43.96
APmedium	88.71	72.88	72.14
APlarge	91.40	74.06	73.74
**COCO Average Recall**			
AR1	16.47	14.24	14.57
AR10	74.56	60.47	61.24
AR100	83.33	66.99	67.16
ARsmall	68.93	49.30	49.62
ARmedium	90.32	76.78	76.33
ARlarge	92.71	77.24	77.31

YOLOv8x6 with an input size of 1280 × 1280 outperforms YOLOv8l with an input size of 640 × 640, exhibiting better results for all metrics, especially for small objects. The training and validation performances were included to demonstrate that there was no overfitting. The models were evaluated on a test set that was not previously encountered during training or validation to assess their generalization capability. The outcomes indicate that both YOLOv8x6 and YOLOv8l attained high APs and ARs for all IoU thresholds and object sizes, except for small objects that are typically distant. Indicating their effectiveness in detecting goats in outdoor environments, especially for monitoring a grazing area. The detections generated by the models are not always perfect and may result in missing parts of the animal, such as the tail, legs or head, or confusing parts between overlapping individuals. These observations are probably not related to the quality of the dataset, but more certainly to the limitation of the models. This may highlight the need for further development and improvement of object detection models.

## Usage Notes

This data paper present the first fine-grained annotated goat dataset for outdoor goat detection in natural environments. The dataset contains a total of 6160 images, captured by a Trecker X2, a CCTV camera, a time-lapse camera and a drone. The images and videos feature different breeds, colours, and genders of goats grazing on different pastures of different sizes, mostly in Guadeloupe, French West Indies. The dataset was carefully annotated by expert, providing ground truth labels for goat detection. This dataset can be used to train and evaluate computer vision algorithms for goat detection and tracking, which can have significant applications in precision livestock farming. The dataset and annotations are made publicly available, with the authors aiming to encourage collaborations and accelerate progress in the field. The authors hope that this dataset will stimulate further studies on animal behavior analysis and serve as a cornerstone for a new generation of computer vision applications in agriculture.

### Implications and potential applications of the dataset

CherryChèvre offers vast potential for applications in precision agriculture, such as monitoring animal welfare, behavior, and health^28^. It can be used to optimize breeding programs, enhance productivity, diagnose and treat medical conditions in livestock and pets^29^. The dataset can also be used to monitor animal welfare in transportation, prevent livestock theft, monitor habitat usage and detect poaching in wildlife conservation^30^. Furthermore, researchers can use it to study animal behavior, cognition, social interaction^31^, and self-medication^32^.

### Limitations and possible improvements of the dataset

The proposed dataset in this paper has few limitations that must be taken into consideration. One of the main limitations is that the dataset was acquired at a specific location, which means that it may not be representative of all natural environments. For example, there are no goats near sea, lake, forest or in a snowy background. Another limitation is that the data set contains few distinct goats, each possessing a unique coat color. While this may be sufficient for most studies, it limits the generalizability of the dataset. Furthermore, the dataset mostly contains outdoor goat images, which means that the learned model may lose accuracy in detecting goats indoors. Within the dataset, some birds are visible (mainly *Bubulcus ibis*, *Corvus corax* and *Quiscalus lugubris*), otherwise no other species are present, thus a detector trained on this dataset may detect other animals, like dogs, cows, horses, cats, etc.

Another issue may be linked to the use of bounding boxes, in some cases, it can be challenging to draw them accurately around animals. The difficulty for the annotator is to determine whether to define the reality seen by a human or the detection expected by the algorithm. Some examples of such cases include:

- Occlusion: Drawing accurate bounding boxes around animals in images can be challenging, especially when the animal’s body is partially hidden behind an object or another animal. This makes it difficult to determine whether the bounding box should include the entire body or be splited into multiple boxes.
- Blurry edges: In some cases, an animal’s body may have fuzzy edges due to fur, motion, shadow, specular or weed, making it difficult to determine the exact boundary of the animal.
- Grouping: In situations where animals are in groups or herds, it can be challenging to separate individual animals and accurately annotate them with bounding boxes. Especially on sheep, as they often huddle closely together and have similar body shapes and sizes, making it difficult to distinguish one from another.

In such cases, alternative annotation methods such as segmentation or keypoint annotations may be more appropriate. Such annotation could add awesome value to the dataset by providing more detailed information about the animal’s behavior and movements. For example, segmentation can help track the movement of individual body parts, which can be valuable for studying animal behavior, locomotion, and biomechanics. Keypoint detection can be used to analyze an animal’s posture, facial expressions, and social interactions, providing insights into their communication and social behavior.

Incorporating a depth order for each annotated bounding box is an additional enhancement that can greatly improve the accuracy of tracking in animal behavior analysis. This enhancement would enable the prediction of overlapping bounding boxes and their relative order, providing valuable information about the spatial relationship between objects in the scene, including which object is in front of the camera when multiple objects overlap.

In terms of improvements, future studies could focus on expanding the dataset by including more diverse environments and goat individuals. Additionally, the dataset could be extended to include additional attributes or labels beyond simple detection, such as age, gender, weight or behavior, to support more complex studies. The author awaiting for collaboration on these subjects.

## Data Availability

The Python 3.10 scripts used for converting the VGG VIA csv format to YOLO format, as well as other scripts used for generating statistics presented in the article, are available at 10.57745/QEZBNA^24^.
